# Assessment for diagnosis of lymph node metastasis in esophageal cancer using endoscopic ultrasound elastography

**DOI:** 10.1007/s10388-016-0521-0

**Published:** 2016-01-29

**Authors:** Tetsutaro Sazuka, Takashi Akai, Masaya Uesato, Daisuke Horibe, Mari Kuboshima, Hiroyuki Kitabayashi, Akinao Matsunaga, Akiko Kagaya, Yorihiko Muto, Nobuyoshi Takeshita, Tetsuro Maruyama, Yukimasa Miyazawa, Kiyohiko Shuto, Toru Shiratori, Tsuguaki Kono, Yasunori Akutsu, Isamu Hoshino, Hisahiro Matsubara

**Affiliations:** Department of Frontier Surgery, Chiba University Graduate School of Medicine, 1-8-1 Inohana, Chuo-ku, Chiba-shi, Chiba, 260-8670 Japan

**Keywords:** Diagnosis, Esophageal cancer, EUS elastography, Lymph node, Metastasis

## Abstract

**Background:**

We performed endoscopic ultrasound real-time tissue elastography to more accurately diagnose lymph node metastasis of esophageal cancer. The aim of this study was to evaluate the ability of EUS elastography to distinguish benign from malignant lymph nodes in esophageal cancer patients.

**Methods:**

The present study had two steps. As the first step (study 1), we developed diagnostic criteria for metastatic lymph nodes using elastography and verified the validity of the criteria. Three hundred and twenty-two lymph nodes from 35 patients treated by surgical resection were included in the study. As the second step (study 2), we preoperatively examined the lymph nodes of esophageal cancer patients with EUS elastography and compared its diagnostic performance with that of the conventional B-mode EUS images. A total of 115 lymph nodes from 31 patients were included.

**Results:**

In study 1, lymph nodes were considered malignant if 50 % or more of the node appeared blue, or if the peripheral part of the lesion was blue and the central part was red/yellow/green. The sensitivity and specificity of the elastography were 79.7 and 97.6 % with an accuracy of 93.8 %, which was significantly higher than the values for conventional B-mode imaging. In study 2, the sensitivity and specificity of the EUS elastography were 91.2 and 94.5 % with an accuracy of 93.9 %, which was also significantly higher than the values for conventional B-mode EUS imaging.

**Conclusions:**

The present study demonstrated that EUS elastography is useful for diagnosing lymph node metastasis of esophageal cancer.

## Introduction

The prognosis of patients with esophageal squamous cell carcinoma (ESCC) is poor. The treatment is determined based on the preoperative tumor stage, with the lymph node stage considered the most important factor [1–3]. We use the diagnosis of lymph node metastasis to determine the treatment options for patients with esophageal cancer. The presence of either five or more positive nodes in any field, or metastatic nodes present in all three (cervical, mediastinal, and abdominal) fields have been considered indication for neoadjuvant chemoradiation therapy at our department [4]. On the other hand, the indications for endoscopic resection of esophageal cancer are limited to cases without lymph node metastasis. Therefore, the nodal status is essential information to determine the treatment strategies for both advanced and early esophageal cancer.

Although, techniques such as computed tomography (CT), magnetic resonance imaging (MRI), fluorine-18 fluorodeoxyglucose positron emission tomography (FDG-PET), and endoscopic ultrasound (EUS) are used to evaluate lymph node metastasis [5, 6], the sensitivities and specificities of those modalities are still not satisfied.

Real-time tissue elastography (elastography) is a new imaging procedure used for the visualization of tissue elasticity which can be applied during ultrasound examination. The technology measures the degree of tissue deformation after compression as an indicator of the stiffness of the tissue. Pathological changes are generally known to be correlated with changes in tissue elasticity. Generally, malignant tumors are harder than benign tumors [7]. The elasticity of a tissue is reconstructed within a sample area and translated into a color signal that overlies the real-time B-mode image. The elasticity of the sample area is classified relatively in 256 levels by elastography. The colors associated with hard, intermediate, and soft tissues are blue, green/yellow, and red, respectively.

Elastography is more useful for the diagnosis of cancer with regard to the visualization of tissue elasticity compared to the conventional B-mode ultrasonography. Endoscopic ultrasound real-time tissue elastography (EUS elastography) offers, as a new type of diagnostic information, the stiffness of the lymph node, allowing for more sophisticated diagnostic performance.

The aim of this study was to evaluate the ability of EUS elastography to distinguish between benign and malignant lymph nodes from patients with esophageal cancer.

## Materials and methods

### Patients and clinical lymph node samples

Lymph nodes (*n* = 322) from 35 ESCC patients treated by surgical resection between October 2009 and August 2011 in the Department of Frontier Surgery, Chiba University Graduate School of Medicine, was included in this study. All lymph nodes visualized by elastography were examined. In preoperative study, 31 patients were examined with EUS elastography for their cervical, mediastinal or abdominal lymph nodes before operation. Written consent for this study was obtained from each patient before examinations.

### Establishment of lymph nodes evaluation using EUS elastography

As the first step (study 1), the lymph nodes of patients with esophageal cancer treated by surgical resection were examined by elastography and conventional B-mode imaging with a free hand linear array transducer before formalin fixation. The description form with elastography was classified, and the diagnostic criteria for metastatic lymph nodes were developed. The ultrasound platform which was used in all studies was an EUB-7500 (Hitachi Medical Systems, Tokyo, Japan). We used a convex EUS probe (EG-3870UTK; Hoya: Pentax division, Tokyo, Japan). The technical details of using these instruments are described elsewhere [8].

We examined the removed lymph node adhered by surrounding tissue (such as fiber or fat). We put each lymph node under the resected esophagus and scan the lymph node through the esophagus wall. Thereby, each lymph node could be visualized ultrasonographically and it was possible to obtain an image substantially similar to the image that has been examined in vivo using EUS elastography. Elastographic and B-mode images were displayed simultaneously side by side. The lymph node characteristics were carefully described for each lymph node: size (long axis), echogeneity (hypoechoic, hyperechoic), shape (round, oval, flat), border (regular, irregular), and elastographic pattern.

We compared the diagnostic performance of elastography with that of the conventional B-mode technique. Lymph nodes were considered malignant if three or more of the following B-mode criteria were present: lymph node >5 mm in width, round shape, hypoechoic pattern, and smooth border [3].

Lymph nodes were considered malignant if 50 % or more of the node appeared blue, or if the peripheral part of lesion was blue and the central part was red/yellow/green. Lymph nodes were considered negative for metastasis if they showed homogeneous green/yellow/red, or a mosaic pattern of green/yellow/red, or less than 50 % of the node was blue. The background of the creation of the diagnostic criteria will be discussed later.

Upon the development of the diagnostic criteria for lymph node metastasis by elastography, we referred to a scoring system based on the elastographic patterns reported in previous studies [9–11].

### Preoperative lymph nodes evaluation by EUS elastography

As the second step (study 2), we preoperatively examined the lymph nodes of ESCC patients by EUS elastography and discriminated between benign and malignant nodes based on the newly-developed diagnostic criteria, and compared diagnostic performance of EUS elastography with that of the conventional B-mode EUS imaging. To　collate the lymph node examined by EUS elastography with the lymph node removed by the operation, we performed the following tasks: we recorded the position, size, and shape of each lymph node in EUS elastography, and at the time of operation, the position of the lymph node was checked, and the size and form of lymph node which was removed was again recorded with a free hand linear array transducer. We considered it the same lymph node whose position was the same, and size and form were most similar. A total of 115 lymph nodes were included. All lymph nodes visualized by EUS elastography were examined.

### Statistical analysis

χ^2^ test or Fisher’s exact test was used to compare diagnostic performance of elastography and B mode images. All calculations were done with SPSS 15.0 (SPSS Inc, Chicago, IL). *P* values of less than 0.05 were considered to be statistically significant.

## Results

### The diagnostic criteria for lymph node metastasis by elastography

**Fig. 1:**
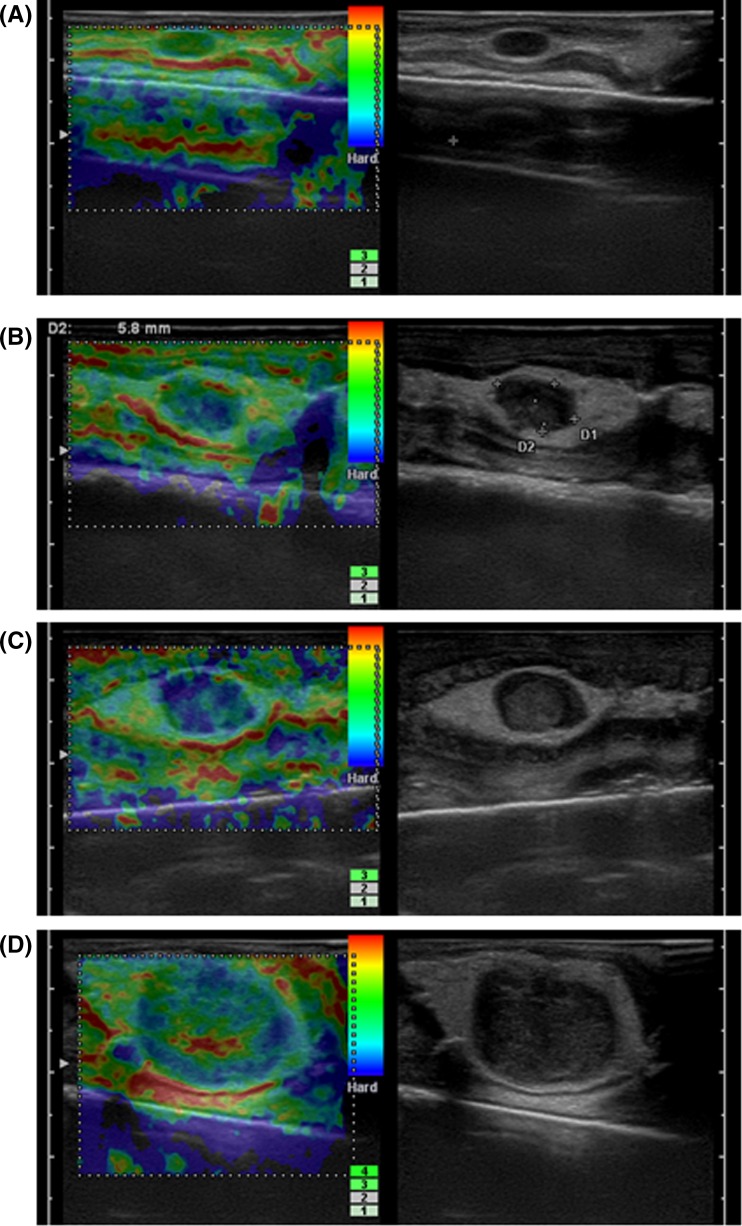
**a** Pattern 1: relatively homogeneous coloring or the areas with two or three different colors (*red*, *yellow*, and/or *green*). **b** Pattern 2: *red*, *yellow*, *green* and *blue* were mixed. **c** Pattern 3: almost the entire area was observed to be *blue*. **d** Pattern 4: the margin of the tumor was depicted in *blue*, and the center was depicted in *red*, *yellow* or/and *green*

We classified the elastographic images into 4 patterns, as follows:

Pattern 1: relatively homogeneous coloring or areas of two or three different colors with red, yellow, green (Fig. 1a)

.

Pattern 2: areas of red, yellow, green and blue were mixed (Fig. 1b).

Pattern 3: almost the entire area was blue (Fig. 1c).

Pattern 4: the margin of the tumor was depicted in blue, and the center in red, yellow or/and green (Fig. 1d).

Although there are minor differences, all of the previous studies indicated that malignancy is suggested by an increased proportion of blue. Pattern 4 seemed to be a characteristic finding that reflected the internal necrosis of cancer. When pattern 1 was assumed to be negative for metastasis, and the rest was assumed to be positive, the negative predictive value was excellent, at 94.0 %. When patterns 3 and 4 were assumed to be positive for metastasis, and 1 and 2 were assumed to be negative, the positive predictive value was excellent, at 95.7 %. Therefore, it seemed to be reasonable to regard that pattern 1 as negative and patterns 3 and 4 as positive findings. However, it was difficult to decide how to subdivide pattern 2 to increase the diagnostic performance.

**Fig. 2:**
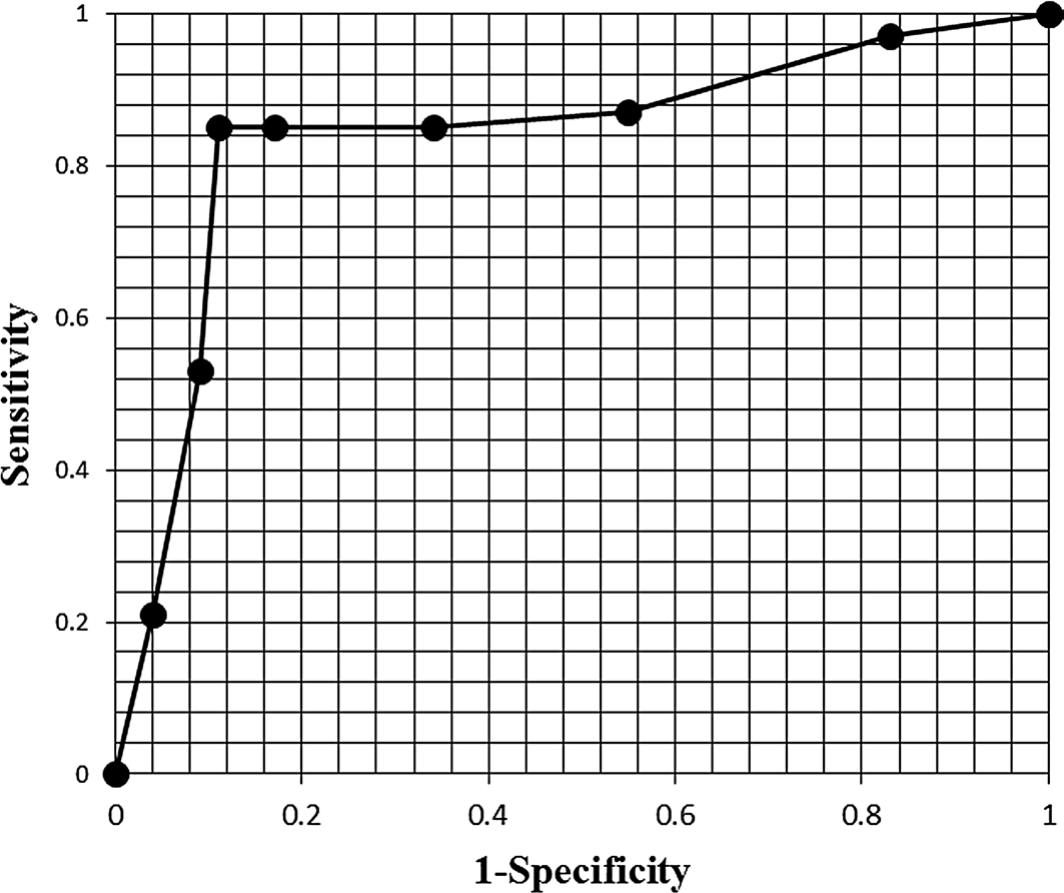
Lymph nodes shown to be pattern 2 by elastography (*N* = 86) were evaluated for their percentage of *blue*, and a receiver operator characteristic curve was generated to determine the optimal cut-off value. This figure shows that if the lymph node is observed to be more than 50 % *blue*, it should be diagnosed as metastasis. This resulted in a sensitivity of 85 % and a specificity of 89 %

To solve this problem, we measured the area of lymph node and the blue area inside as a circle or ellipse for every lymph node shown to be pattern 2, evaluated the percentage of blue, and a receiver operator characteristic curve was generated to determine the cut off value (Fig. 2). If the cut-off value was assumed to be 50 %, the sensitivity was 85 % and the specificity was 89 %.

**Fig. 3:**
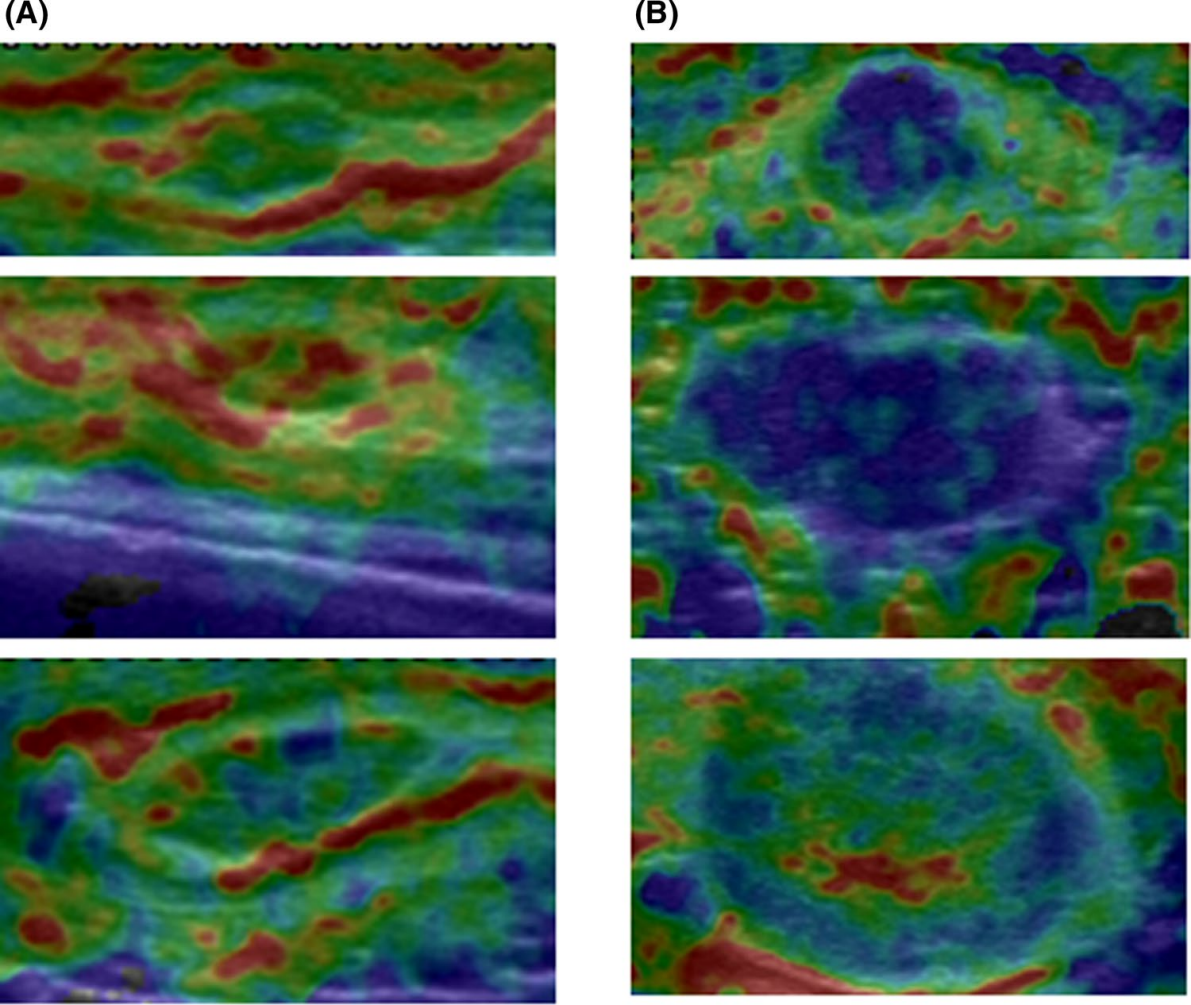
Diagnostic criteria for metastatic lymph nodes by elastography are shown. Lymph nodes were considered negative for metastasis if showed homogeneous *green*/*yellow*/*red*, or a mosaic pattern of *green*/*yellow*/*red*, or less than 50 % of the node was *blue* (**a**). Lymph nodes were considered malignant if 50 % or more of the node appeared *blue*, or the peripheral part of lesion was *blue* and the central part was *red*/*yellow*/*green* (**b**)

Therefore, the lymph nodes that were observed to be 50 % or more blue were assumed to be metastasis positive in this study. In other words, the criteria for diagnosing negative lymph nodes using elastography were the image like pattern 1 and 2, and the image which the blue part of a lymph node was less than 50 % (Fig. 3a), and the criteria for diagnosing positive lymph nodes were the presence of blue accountings for 50 % or more of the lesion (including pattern 3), and lesions characterized by pattern 4 (Fig. 3b).

**Fig. 4:**
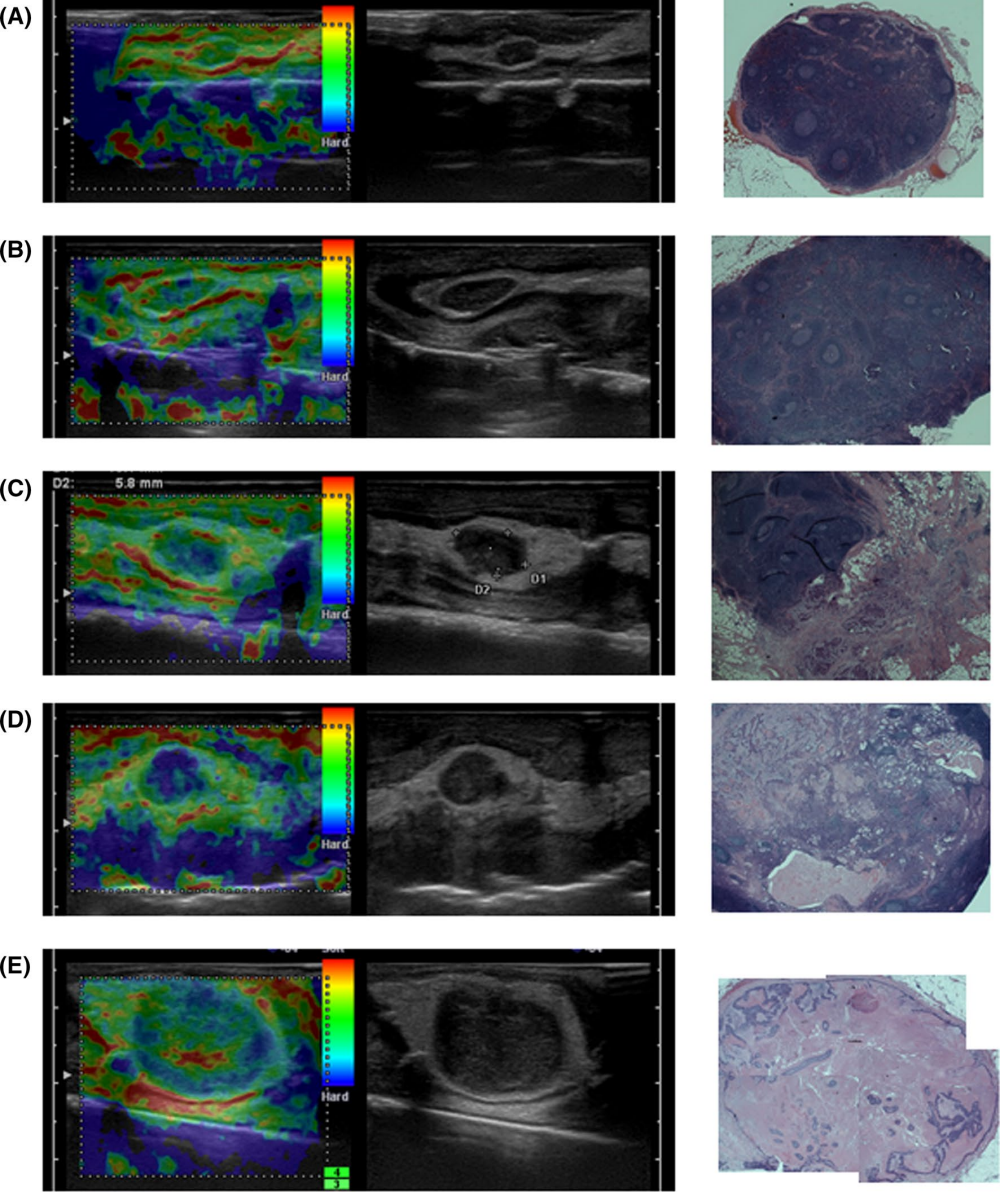
Typical images are shown. The elastographic images are shown in upper stage, lower is pathological each. **a** A sample which was shown to be *green* by elastography and proved to be negative for metastasis. **b** A sample which *blue dots* were seen but the percentage compared with the whole area of the lymph node was small, and it was pathologically negative. **c** A sample which the *blue* ratio among the lymph node is more than 50 % and proved to be positive for metastasis. Localization of *blue* indicated by elastography and localization of metastatic focus in pathological finding appears to be similar. **d** A sample which was shown to be *blue* and proved to be metastatic-positive. The occupation rate was 100 %. **e** A sample whose border of the lymph node was *blue*, and the inside was depicted with *green*, *red*, and *yellow*. It was pathologically positive for metastasis accompanied by necrosis inside

Typical images are shown in Fig. 4. The elastographic images are shown in upper stage, lower is pathological each. (a) A pattern 1 sample which was shown to be green by elastography and proved to be negative for metastasis. (b) A pattern 2 sample which blue dots were seen but the percentage compared with the whole area of the lymph node was small, and it was pathologically negative. (c) Also pattern 3 sample which the blue ratio among the lymph node is more than 50 % and proved to be positive for metastasis. Localization of blue indicated by elastography and localization of metastatic focus in pathological finding appears to be similar. (d) Also pattern 3 sample which was shown to be blue and proved to be metastatic positive. The occupation rate was 100 %. (e) A pattern 4 sample whose border of the lymph node was blue, and the inside was depicted with green, red, and yellow. It was pathologically positive for metastasis accompanied by necrosis inside.

### Accuracy of the elastography in lymph node evaluation

**Fig. 5:**
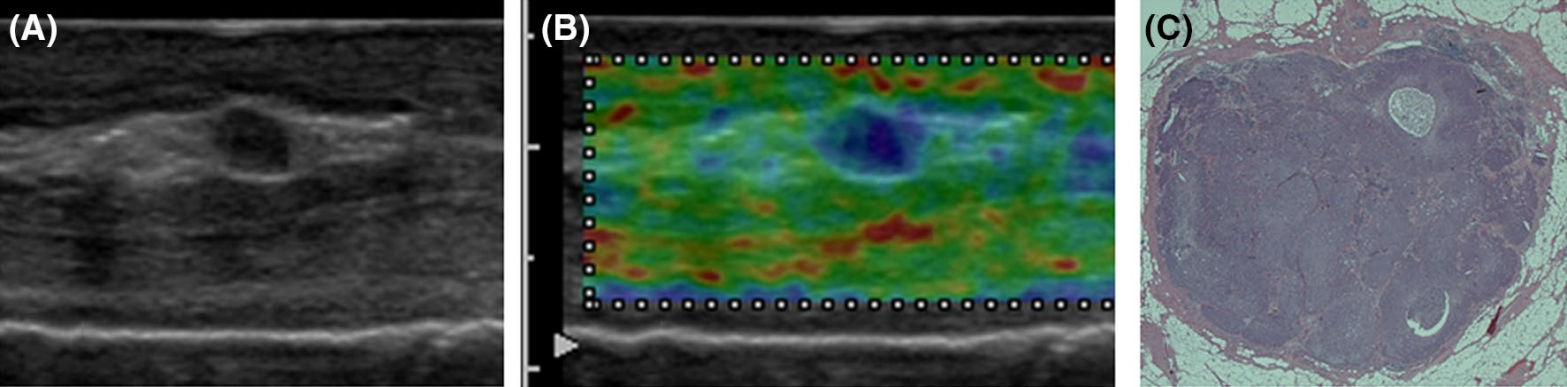
The lymph node was 4 × 3.1 mm, round shaped, had a smooth border, and the internal echo was homogenous in B mode (**a**). The entire lymph node was shown to be *blue* by elastography, and the lymph node was classified into Pattern 3 (**b**). The pathological examination revealed that the almost the entire lymph node was full of carcinoma cells (**c**)

Histological examination of the surgical specimens revealed benign tissue in 253 lymph nodes, and malignant tissue in 69 lymph nodes. The elastographic images were interpreted as benign in 247 lymph nodes, and malignant in 55 lymph nodes. The calculated sensitivity, specificity, positive and negative predictive values of the elastography were 79.7, 97.6, 90.2 and 94.6 %, respectively, with an accuracy of 93.8 %. The calculated sensitivity, specificity, positive and negative predictive values for the conventional B-mode images were 62.3, 60.6, 30.3 and 85.6 %, respectively, with an accuracy of 61.1 %. The sensitivity and specificity of elastography were significantly better than those of the B-mode technique (Table 1). A detailed investigation revealed that the smaller metastatic lymph node can be detected by elastography compared with B-mode. A representative case is shown in Fig. 5. The lymph node was 4 × 3.1 mm, round shape, smooth border, an internal echo was homogenous in B mode (Fig. 5a), and it was diagnosed as negative. On the other hand, the entire lymph node was shown to be blue by elastography, and the lymph node was classified as having Pattern 3, and was therefore diagnosed as positive (Fig. 5b). The pathological findings revealed that the whole lymph node was almost completely occupied by the carcinoma cells (Fig. 5c).

**Table 1:** Elastography versus B mode image for diagnosis of lymph node metastasis

	Sensitivity	Specificity	Accuracy
Elastography (%)	79.7 (55/69)	97.6 (247/253)	93.8 (302/322)
B mode (%)	62.3 (43/69)	60.6 (154/253)	61.1 (197/322)
*P* value elastography vs. B mode	0.02	<0.01	<0.01

The results of the histological examination of the surgical specimens revealed benign tissue in 253 lymph nodes, and malignant tissue in 69 lymph nodes. The elastographic images were interpreted as benign in 247 samples, and malignant in 55 samples. The sensitivity and specificity of elastography were significantly better than those of B-mode imaging

In study 2, the histological examination of the surgical specimens revealed benign tissue in 91 lymph nodes, and malignant tissue in 24 lymph nodes. The images of EUS elastography were interpreted as benign in 86 lymph nodes, and malignant in 22 lymph nodes. The calculated sensitivity, specificity, positive and negative predictive values of the EUS elastography were 91.2, 94.5, 81.5 and 97.8 %, respectively, with an accuracy of 93.9 %. The calculated sensitivity, specificity, positive and negative predictive values of the conventional B-mode EUS were 62.5, 73.5, 38.5 and 88.2 %, respectively, with an accuracy of 71.3 %. The sensitivity and specificity of EUS elastography were significantly better than those of the B-mode technique (Table 2).

**Table 2:** EUS elastography versus B mode EUS image for diagnosis of lymph node metastasis

	Sensitivity	Specificity	Accuracy
EUS elastography (%)	91.2 (22/24)	94.5 (86/91)	93.9 (108/115)
B mode EUS (%)	62.5 (15/24)	73.5 (67/91)	71.3 (82/115)
*P* value EUS elastography vs. B mode EUS	0.04	<0.01	<0.01

The results of the histological examination of the surgical specimens revealed benign tissue in 91 lymph nodes, and malignant tissue in 24 lymph nodes. The images of EUS elastography were interpreted as benign in 86 samples, and malignant in 22 samples. The sensitivity and specificity of EUS elastography were significantly better than those of B-mode imaging

In study 2, with since the observation of anal side of the tumor which is making stenosis is difficult, and the lymph node at the position away from gastrointestinal tract cannot be targeted for inspection, it is thought that there were fewer evaluation lymph nodes in one case in Study2 than Study1. The compression during EUS examination is naturally obtained by arterial pulsations and respiratory movements [12].

## Discussion

It is important to comprehensively examine and fully understand the advantages and disadvantages of each modality that can be used to diagnose metastasis from esophageal cancer. Van Vliet et al. reported that the random effects pooled sensitivities of EUS, CT, and FDG-PET for regional lymph node metastases were 0.80 (95 % confidence interval 0.75–0.84), 0.50 (0.41–0.60), and 0.57 (0.43–0.70), respectively, and the specificities were 0.70 (0.65–0.75), 0.83 (0.77–0.89), and 0.85 (0.76–0.95), respectively [13]. Sakurada et al. reported that the average patient-based sensitivity and specificity for the detection of lymph-node metastasis, using the diffusion-weighted whole-body magnetic resonance imaging with background body signal suppression (DWIBS) sequence, were 77.8 and 55.6 % [6].

In our study, EUS elastography showed tremendous accuracy with high sensitivity and specificity. Although EUS cannot be used in cases of stenosis, and there are blind areas (such as around the trachea) associated with the use of EUS, EUS allows for detailed observation of one area and so can provide a lot of information about that area. In addition, it is not affected by the pulsation of the heart and aorta, so it is considered to be the most accurate modality for the diagnosis of lymph node metastasis. EUS elastography, as a new diagnostic strategy, evaluates the stiffness of the tissue as a means of tissue characterization, and more sophisticated diagnostic performance can be expected from this technique.

It is well recognized in the literature that the elastic properties of a tissue provide the means for not only characterizing that tissue, but also for differentiating normal from diseased tissues. This conclusion is based on a wealth of data obtained in studies performed on excised breast specimens [14, 15] and clinical studies of various conditions conducted by numerous researchers worldwide [9, 16, 17].

Ultrasound elastography has been developed into an effective imaging method that can be used to estimate the local elastic properties of biological tissues [7]. Typically, an external, static, or quasi-static, compression is applied on the tissue. The resulting displacement distribution within the tissue is estimated using a cross-correlation analysis on the acquired pre- and post-compression radio-frequency (RF) signals. The strain distribution is then calculated as the spatial gradient of the displacement [7, 18]. By solving the inverse problem, the shear modulus, or Young’s modulus, can be reconstructed from the estimated displacement and strain images, i.e., elastograms [19]. Several different elastographic methods and applications have been developed over the past few decades. For example, elastography has been successfully applied for the diagnosis of breast lesions and is currently used in the clinic [9, 20].

Studies using EUS elastography were first reported by Giovannni et al. [10]. Many of the studies using EUS elastography have been reported on the pancreas and lymph nodes, although there have also been a few reports on the gastrointestinal tract and liver [12, 21–30].

**Fig. 6:**
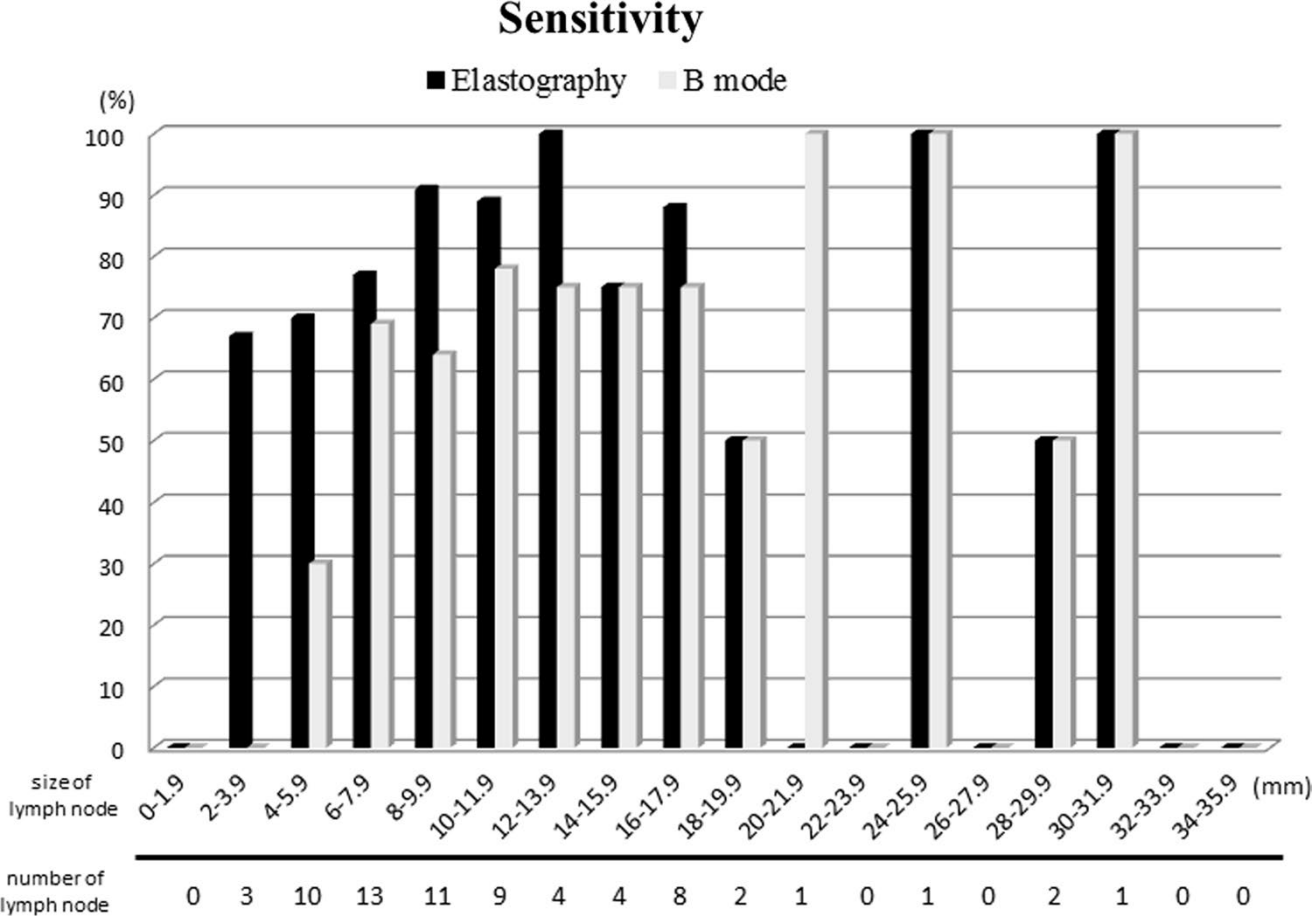
The sensitivity of elastography and B mode imaging was compared by dividing the lymph nodes in 2 mm segments along the major axis. The sensitivity of each size is shown as a *bar graph*. The sensitivity of elastography is shown as *black bar*, and B mode is shown as *white bar*. In the lymph nodes smaller than 6 mm, the sensitivity of elastography tended to be higher than that of B mode imaging

Study 1 revealed that the sensitivity of elastography was significantly better than those of the B-mode technique. In order to investigate the reason for the improved performance, the sensitivity of elastography and B mode imaging was compared by dividing the lymph nodes in 2 mm segments along the major axis (Fig. 6). The sensitivity of each size is shown as a bar graph. The sensitivity of elastography is shown as black bar, and B mode is shown as white bar.

As shown in the graph, the sensitivity of elastography tended to be higher than that of B mode in the lymph node smaller than 6 mm. The smallest size detected by elastography was 3.4 mm in the major axis. The occupation rate was 100 %. Thus, elastography can detect small metastatic lymph nodes which could not detect previously by the other modality [31]. To make more precise criteria for metastatic lymph node, we think it is necessary to investigate the micrographics in detail and to compare the blue area of elastographic image and the metastatic lesion of a lymph node. This is the next task.

**Fig. 7:**
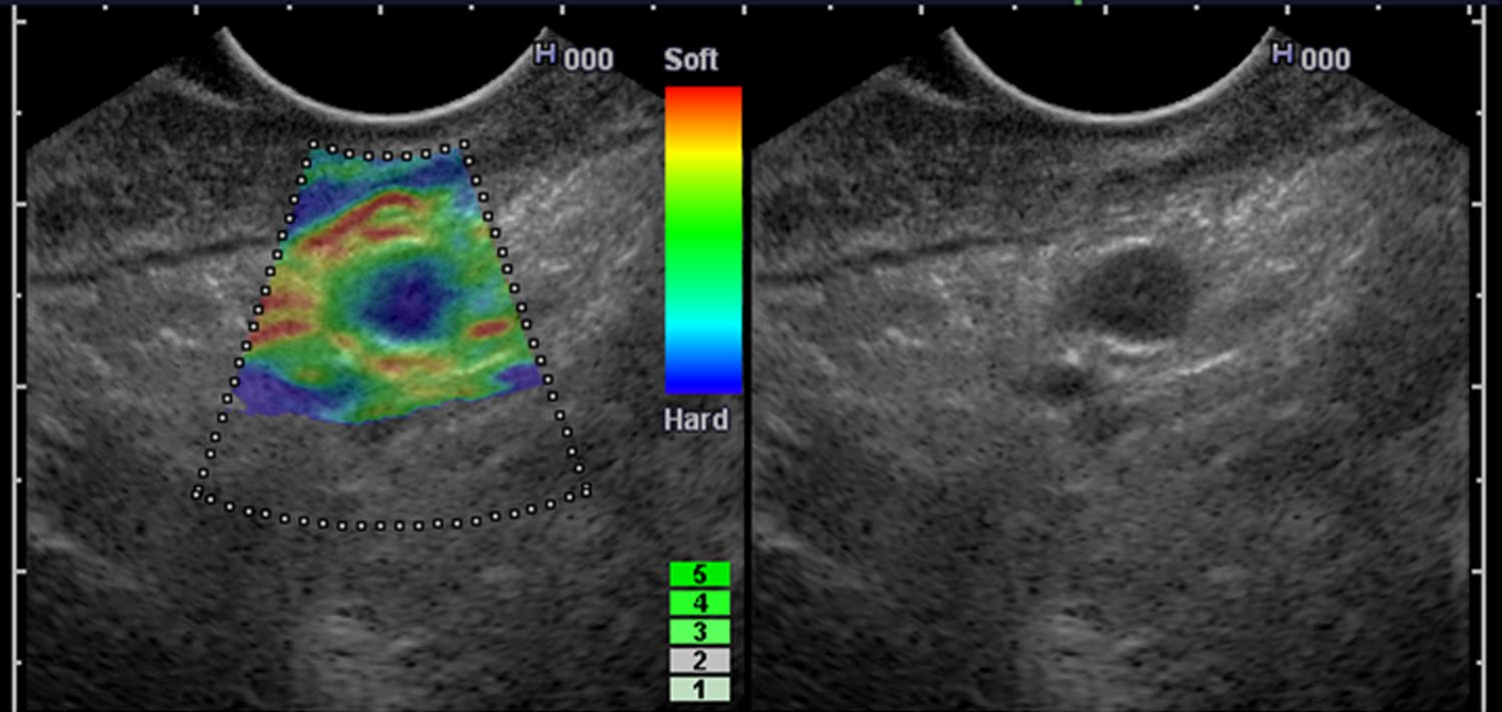
The imaging of EUS elastography is almost the same as that of elastography with a free hand linear array transducer

In study 2, EUS elastography was used preoperatively to examine the lymph nodes. The diagnostic performance of EUS elastography was compared with that of the B mode of EUS. The imaging of EUS elastography is almost the same as that of elastography with a free hand linear array transducer (Fig. 7). Therefore, it seemed to be reasonable to regard that the newly-developed diagnostic criteria can be used for EUS elastography.

**Fig. 8:**
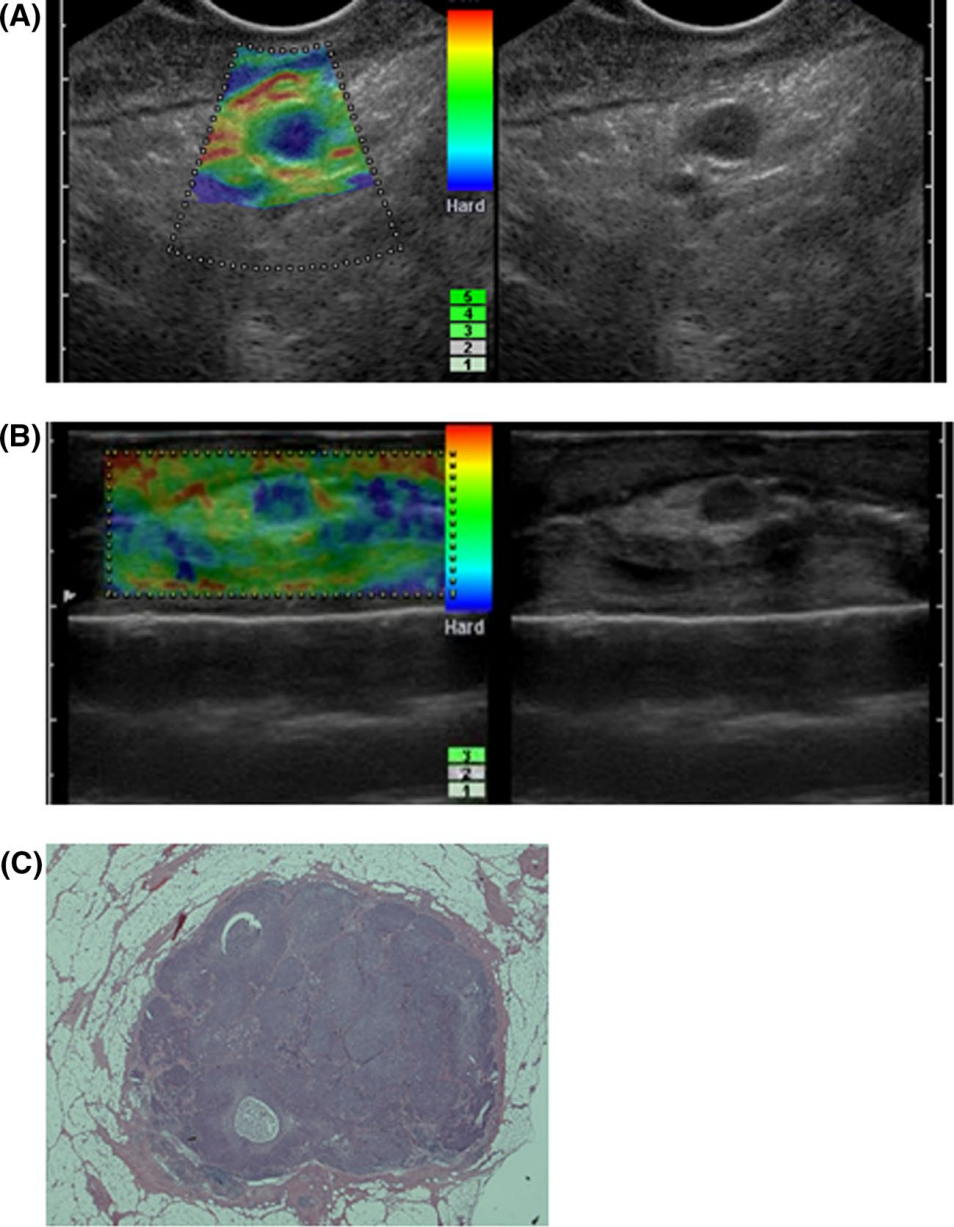
A left recurrent nerve lymph node was detected by EUS elastography (**a**). The lymph node was round and the size was 6.6 × 6.0 mm. It was shown to be almost *blue* and classified as pattern 3. The resected lymph node was examined by free hand probe (**b**). It was round shape and the size was 6.3 × 5.5 mm. It was classified as pattern 3. Pathological examination revealed that the lymph node was metastatic one (**c**)

Representative images are shown in Fig. 8. A left recurrent nerve lymph node was detected by EUS elastography (Fig. 8a). The lymph node was round and the size was 6.6 × 6.0 mm. It was shown to be almost blue and classified as pattern 3. The resected lymph node was examined by free hand probe (Fig. 8b). It was round shape and the size was 6.3 × 5.5 mm. It was classified as pattern 3. Pathological examination revealed that the lymph node was metastatic one (Fig. 8c). It was 6 mm in diameter. It seemed that the image of free hand probe tended to become an oval or the flat form and we think the reason is because the pressure of free hand probe tended to be stronger than that of EUS probe.

In the examination with EUS, the sensitivity and the specificity of elastography were significantly better compared with B mode images, and it was suggested that the technique could contribute to the preoperative diagnosis of the patient. The present study demonstrated that EUS elastography could be useful for the preoperative diagnosis of lymph node metastases of esophageal cancer.

## References

[1] Walsh TN, Noonan N, Hollywood D, Kelly A, Keeling N, Hennessy TP (1996). A comparison of multimodal therapy and surgery for esophageal adenocarcinoma. N Engl J Med.

[2] Fockens P, Kisman K, Merkus MP, van Lanschot JJ, Obertop H, Tytgat GN (1998). The prognosis of esophageal carcinoma staged irresectable (T4) by endosonography. J Am Coll Surg.

[3] Vazquez-Sequeiros E, Wiersema MJ, Clain JE, Norton ID, Levy MJ, Romero Y (2003). Impact of lymph node staging on therapy of esophageal carcinoma. Gastroenterology.

[4] Nabeya Y, Ochiai T, Matsubara H, Okazumi S, Shiratori T, Shuto K (2005). Neoadjuvant chemoradiotherapy followed by esophagectomy for initially resectable squamous cell carcinoma of the esophagus with multiple lymph node metastasis. Dis Esophagus.

[5] van Vliet EP, Heijenbrok-Kal MH, Hunink MG, Kuipers EJ, Siersema PD (2008). Staging investigations for oesophageal cancer: a meta-analysis. Br J Cancer.

[6] Sakurada A, Takahara T, Kwee TC, Yamashita T, Nasu S, Horie T (2009). Diagnostic performance of diffusion-weighted magnetic resonance imaging in esophageal cancer. Eur Radiol.

[7] Ophir J, Cespedes I, Ponnekanti H, Yazdi Y, Li X (1991). Elastography: a quantitative method for imaging the elasticity of biological tissues. Ultrason Imaging.

[8] Frey H (2003). Realtime elastography. A new ultrasound procedure for the reconstruction of tissue elasticity. Radiologe.

[9] Itoh A, Ueno E, Tohno E, Kamma H, Takahashi H, Shiina T (2006). Breast disease: clinical application of US elastography for diagnosis. Radiology.

[10] Giovannini M, Hookey LC, Bories E, Pesenti C, Monges G, Delpero JR (2006). Endoscopic ultrasound elastography: the first step towards virtual biopsy? Preliminary results in 49 patients. Endoscopy.

[11] Furukawa MK, Kubota A, Hanamura H, Furukawa M (2007). Clinical application of real-time tissue elastography to head and neck cancer–evaluation of cervical lymph node metastasis with real-time tissue elastography. Nippon Jibiinkoka Gakkai Kaiho.

[12] Giovannini M, Thomas B, Erwan B, Christian P, Fabrice C, Benjamin E (2009). Endoscopic ultrasound elastography for evaluation of lymph nodes and pancreatic masses: a multicenter study. World J Gastroenterol.

[13] van Vliet EPM, Heijenbrok-Kal MH, Hunink MGM, Kuipers EJ, Siersema PD (2008). Staging investigations for oesophageal cancer: a meta-analysis. Br J Cancer.

[14] Skovorda AR, Klishko AN, Gusakian DA, Maevskii EI, Ermilova VD, Oranskaia GA (1995). Quantitative analysis of mechanical characteristics of pathologically altered soft biological tissues. Biofizika.

[15] Krouskop TA, Wheeler TM, Kallel F, Garra BS, Hall T (1998). Elastic moduli of breast and prostate tissues under compression. Ultrason Imaging.

[16] Thomas A, Fischer T, Frey H, Ohlinger R, Grunwald S, Blohmer JU (2006). Real-time elastography–an advanced method of ultrasound: first results in 108 patients with breast lesions. Ultrasound Obstet Gynecol.

[17] Burnside ES, Hall TJ, Sommer AM, Hesley GK, Sisney GA, Svensson WE (2007). Differentiating benign from malignant solid breast masses with US strain imaging. Radiology.

[18] Luo J, Bai J, He P, Ying K (2004). Axial strain calculation using a low-pass digital differentiator in ultrasound elastography. IEEE Trans Ultrason Ferroelectr Freq Control.

[19] Kallel F, Bertrand M (1996). Tissue elasticity reconstruction using linear perturbation method. IEEE Trans Med Imaging.

[20] Hiltawsky KM, Kruger M, Starke C, Heuser L, Ermert H, Jensen A (2001). Freehand ultrasound elastography of breast lesions: clinical results. Ultrasound Med Biol.

[21] Giovannini M (2011). Endoscopic ultrasound elastography. Pancreatology.

[22] Itokawa F, Itoi T, Sofuni A, Kurihara T, Tsuchiya T, Ishii K (2011). EUS elastography combined with the strain ratio of tissue elasticity for diagnosis of solid pancreatic masses. J Gastroenterol.

[23] Saftoiu A, Vilmann P, Gorunescu F, Janssen J, Hocke M, Larsen M (2011). Accuracy of endoscopic ultrasound elastography used for differential diagnosis of focal pancreatic masses: a multicenter study. Endoscopy.

[24] Dietrich CF, Hirche TO, Ott M, Ignee A (2009). Real-time tissue elastography in the diagnosis of autoimmune pancreatitis. Endoscopy.

[25] Iglesias Garcia J, Larino Noia J, Souto R, Alvarez Castro A, Cigarran B, Dominguez Munoz JE. Endoscopic ultrasound (EUS) elastography of the liver. Rev Esp Enferm Dig 2009, 101:717–719. 10.4321/s1130-01082009001000007 19899940

[26] Mishra G, Conway JD (2009). Endoscopic ultrasound in the evaluation of radiologic abnormalities of the liver and biliary tree. Curr Gastroenterol Rep.

[27] Rustemovic N, Cukovic-Cavka S, Opacic M, Petrovecki M, Hrstic I, Radic D (2010). Endoscopic ultrasound elastography as a method for screening the patients with suspected primary sclerosing cholangitis. Eur J Gastroenterol Hepatol.

[28] Janssen J, Dietrich CF, Will U, Greiner L (2007). Endosonographic elastography in the diagnosis of mediastinal lymph nodes. Endoscopy.

[29] Rustemovic N, Opacic M, Cukovic-Cavka S (2009). Endoscopic ultrasonography elastography in gastroenterology. Acta Med Croatica.

[30] Saftoiu A, Vilmann P, Hassan H, Gorunescu F (2006). Analysis of endoscopic ultrasound elastography used for characterisation and differentiation of benign and malignant lymph nodes. Ultraschall Med.

[31] Schroder W, Baldus SE, Monig SP, Beckurts TK, Dienes HP, Holscher AH (2002). Lymph node staging of esophageal squamous cell carcinoma in patients with and without neoadjuvant radiochemotherapy: histomorphologic analysis. World J Surg.

